# Structure−Activity Relationship (SAR) Study of *trans*-Cinnamic Acid and Derivatives on the Parasitic Weed *Cuscuta campestris*

**DOI:** 10.3390/plants12040697

**Published:** 2023-02-04

**Authors:** Antonio Moreno-Robles, Antonio Cala Peralta, Jesús G. Zorrilla, Gabriele Soriano, Marco Masi, Susana Vilariño-Rodríguez, Alessio Cimmino, Mónica Fernández-Aparicio

**Affiliations:** 1 Department of Plant Breeding, Institute for Sustainable Agriculture (IAS), CSIC, Avenida Menéndez Pidal s/n, 14004 Córdoba, Spain; 2 Allelopathy Group, Department of Organic Chemistry, Facultad de Ciencias, Institute of Biomolecules (INBIO), University of Cadiz, C/Avenida República Saharaui, s/n, 11510 Puerto Real, Spain; 3 Department of Chemical Sciences, University of Naples Federico II, Complesso Universitario Monte S. Angelo, Via Cintia, 80126 Naples, Italy; 4 ALGOSUR S.A., Ctra Lebrija-Trebujena km 5.5, 41740 Lebrija-Sevilla, Spain

**Keywords:** enhanced activity, field dodder, growth inhibition, natural compounds, parasitic weeds, structural analogs, sustainable crop protection

## Abstract

*Cuscuta campestris* Yunck. is a parasitic weed responsible for severe yield losses in crops worldwide. The selective control of this weed is scarce due to the difficult application of methods that kill the parasite without negatively affecting the infected crop. *trans*-Cinnamic acid is secreted by plant roots naturally into the rhizosphere, playing allelopathic roles in plant–plant communities, although its activity in *C. campestris* has never been investigated. In the search for natural molecules with phytotoxic activity against parasitic weeds, this work hypothesized that *trans*-cinnamic acid could be active in inhibiting *C. campestris* growth and that a study of a series of analogs could reveal key structural features for its growth inhibition activity. In the present structure–activity relationship (SAR) study, we determined in vitro the inhibitory activity of *trans*-cinnamic acid and 24 analogs. The results showed that *trans*-cinnamic acid’s growth inhibition of *C. campestris* seedlings is enhanced in eight of its derivatives, namely hydrocinnamic acid, 3-phenylpropionaldehyde, *trans*-cinnamaldehyde, *trans*-4-(trifluoromethyl)cinnamic acid, *trans*-3-chlorocinnamic acid, *trans*-4-chlorocinnamic acid, *trans*-4-bromocinnamic acid, and methyl *trans*-cinnamate. Among the derivatives studied, the methyl ester derivative of *trans*-cinnamic acid was the most active compound. The findings of this SAR study provide knowledge for the design of herbicidal treatments with enhanced activity against parasitic weeds.

## 1. Introduction

Parasitic weeds have the capacity to live heterotrophically, extracting their needed water and nutrients from crop vasculature using haustorial connections [1]. The consequent severe yield losses are difficult to control by means of conventional weed management strategies due to the permanent physical connections that characterize their parasitic life forms. In addition, the high fecundity, embryo longevity, seed dormancy, and easy dissemination create persistent parasitic weed seedbanks rendering infested agricultural lands uncultivable for decades [2]. As a consequence, the parasitic weed problem has become a threat to food security [3]. Among parasitic weeds, the dodders contain over 170 *Cuscuta* species distributed across tropical, subtropical, and temperate regions [4,5]. One of the most damaging *Cuscuta* species is field dodder (*Cuscuta campestris* Yunck.), which severely affects the yield of economically important crops worldwide [6]. There is no effective *C. campestris* control for most affected crops [7,8]. *Cuscuta* plants have neither roots nor leaves and obtain their nutrition by infecting crop stems shortly after germination. In the absence of crop attachment, *C. campestris* seedlings die within 7 to 10 days after germination [9]. The identification of natural compounds that inhibit the growth of pre-attached *C. campestris* seedlings is an obvious target in the design of efficient and sustainable parasitic weed control programs [2].

Cinnamic acid is a monocarboxylic acid with the formula C_9_H_8_O_2_, consisting of an acrylic acid with a phenyl substituent at the 3-position. Plants produce cinnamic acid in the form of two isomers, *trans*- and *cis*-isomers [10,11]. *trans*-Cinnamic acid originates from the shikimic acid pathway through the deamination of phenylalanine by l-phenylalanine ammonia-lyase. *trans*-Cinnamic acid is abundant in plants and its hydroxylation to *p*-coumaric acid leads to a plethora of secondary metabolites formed along the phenylpropanoid pathway [12,13,14,15], many of them involved in activities influencing allelopathic interactions between plants [16]. *trans*-Cinnamic acid is also secreted from plant roots into the rhizosphere where it is reported to be involved in allelopathic interactions between plants [17,18,19]. The photoisomerization in plants of *trans*-cinnamic acid leads to the *cis*-isomer of cinnamic acid [11]. In contrast to the *trans*-isomer, the *cis*-cinnamic acid is present in plants only in trace amounts and it is not challenged into the phenylpropanoid pathway, but, instead, it acts independently in other activities influencing plant growth and development [10,20,21,22,23]. Given the allelopathic properties of cinnamic acid described in the literature, we investigated its activity in the parasitic weed *C. campestris* and performed a structure–activity relationship study to discover the effects of different structural features on its growth-inhibitory activity. This SAR study aims to provide knowledge for the design of new herbicides based on natural compounds that could contribute to the development of sustainable crop protection strategies against parasitic weeds.

## 2. Results and Discussion

**Figure 1:**
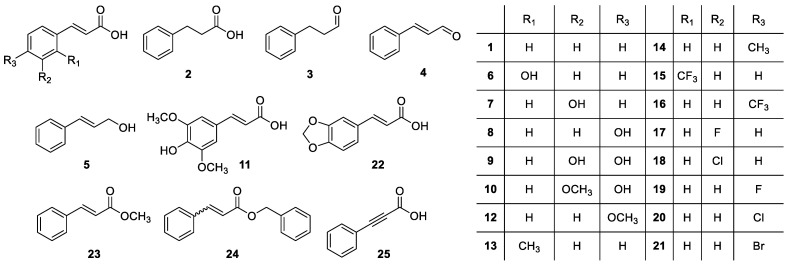
Chemical structures of the compounds studied: *trans*-cinnamic acid (**1**), hydrocinnamic acid (**2**), 3-phenylpropionaldehyde (**3**), *trans*-cinnamaldehyde (**4**), *trans*-cinnamyl alcohol (**5**), *trans*-*o*-coumaric acid (**6**), *trans*-*m*-coumaric acid (**7**), *trans*-*p*-coumaric acid (**8**), *trans*-caffeic acid (**9**), *trans*-ferulic acid (**10**), *trans*-sinapic acid (**11**), *trans*-4-methoxycinnamic acid (**12**), *trans*-2-methylcinnamic acid (**13**), *trans*-4-methylcinnamic acid (**14**), *trans*-2-(trifluoromethyl)cinnamic acid (**15**), *trans*-4-(trifluoromethyl)cinnamic acid (**16**), *trans*-3-fluorocinnamic acid (**17**), *trans*-3-chlorocinnamic acid (**18**), *trans*-4-fluorocinnamic acid (**19**), *trans*-4-chlorocinnamic acid (**20**), *trans*-4-bromocinnamic acid (**21**), *trans*-3,4-(methylenedioxy)cinnamic acid (**22**), methyl *trans*-cinnamate (**23**), benzylcinnamate (**24**), and phenylpropiolic acid (**25**).

**Figure 2:**
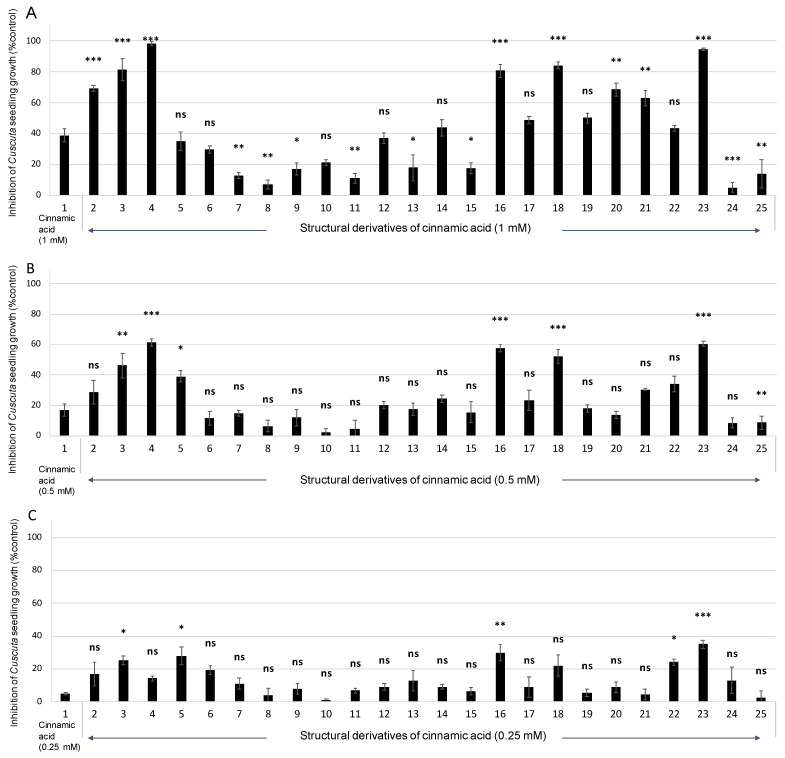
In vitro assessment of the *Cuscuta campestris* growth inhibition induced by compounds *trans*-cinnamic acid (**1**) and derivatives (**2**–**25**) at concentrations of 1 mM (**A**), 0.5 mM (**B**), and 0.25 mM (**C**). Analysis of variance was applied to replicate data, and the *C. campestris* growth inhibition induced by cinnamic acid and derivatives was assessed by the two-sided Dunnett’s test. For each concentration, *, **, and *** indicate significant differences between each treatment with *trans*-cinnamic acid (**1**) at *p* < 0.05, 0.01, and 0.001, respectively; ns indicates no significant difference when comparing each treatment with *trans*-cinnamic acid (**1**). Error bars represent the standard error of the mean.

**Figure 3:**
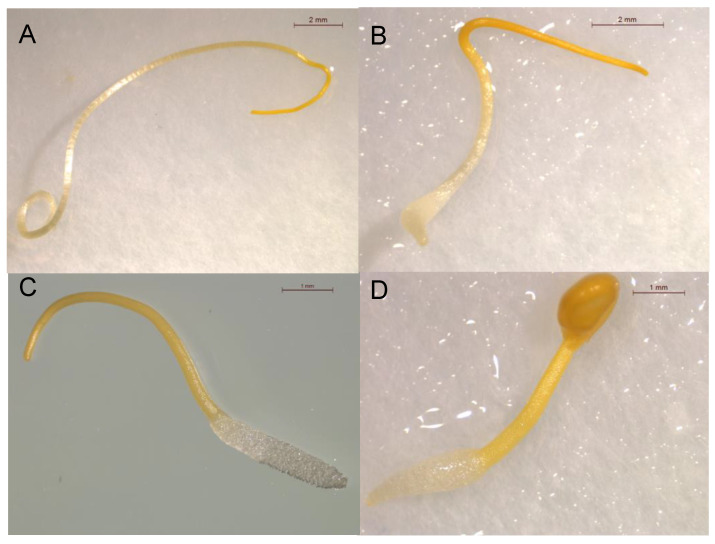
Photographs showing *Cuscuta campestris* seedlings treated with (**A**) a negative control or 1 mM treatments of (**B**) *trans*-cinnamic acid (**1**), (**C**) hydrocinnamic acid (**2**), and (**D**) *trans*-4-bromocinnamic acid (**21**).

The inhibitory activity of *trans*-cinnamic acid (**1**, Figure 1) against the seedling growth of *C. campestris* was studied in vitro. *trans*-Cinnamic acid inhibited growth by 38.9 ± 4.3% in comparison with *C. campestris* seedlings treated with the negative control when tested at 1 mM (Figure 2 and Figure 3A–B). This growth inhibition activity in *C. campestris* seedlings agrees with the growth inhibition reported in lettuce by Hiradate et al. [24]. In order to discover structural features relevant to impart this activity, 24 structural analogs (**2**–**25**, Figure 1) were tested on *C. campestris* growth in comparison with the parent compound, *trans*-cinnamic acid (**1**).

The in vitro study of *trans*-cinnamic acid (**1**) and its analogs (**2**–**25**) revealed that the growth inhibition activity was dependent on the compound treatment (ANOVA, *p* < 0.001). This study allowed the identification of a group of compounds with more pronounced activity than *trans*-cinnamic acid. This group was formed by hydrocinnamic acid (**2**), 3-phenylpropionaldehyde (**3**), *trans*-cinnamaldehyde (**4**), *trans*-4-(trifluoromethyl)cinnamic acid (**16**), *trans*-3-chlorocinnamic acid (**18**), *trans*-4-chlorocinnamic acid (**20**), *trans*-4-bromocinnamic acid (**21**), and methyl *trans*-cinnamate (**23**). In comparison with compound **1,** the study also revealed decreased growth-inhibitory activity in the compounds *trans*-*m*-coumaric acid (**7**), *trans*-*p*-coumaric acid (**8**), *trans*-caffeic acid (**9**), *trans*-sinapic acid (**11**), *trans*-2-methylcinnamic acid (**13**), *trans*-2-(trifluoromethyl)cinnamic acid (**15**), benzylcinnamate (**24**), and phenylpropiolic acid (**25**) (Figure 2).

The study of compounds **1**–**25** on *C. campestris* seedlings in the range of concentrations 0.25–1 mM revealed that the growth-inhibitory activity was concentration-dependent (ANOVA, *p* < 0.001) and that the interaction of compound x concentration was also statistically significant (ANOVA, *p* < 0.001). Higher growth inhibition rates were associated with higher concentrations of the applied compounds in all compounds except for **5**–**11**, **13**, **15**, **24**, and **25**. The growth inhibition results shown in Figure 2 were used to calculate the IC_50_ values in order to compare the effect of the substitution on the bioactivity, and CLog*P* values were calculated to correlate the activity level with the lipophilicity. These parameters are shown in Table 1. The strongest bioactivity was found for compounds **3**, **4**, **16**, **18**, and **23**, with IC_50_ values less than 500 µM. All of them caused at least 80% inhibition at the highest concentration tested (1 mM), which decreased to around 45–60% when the concentration was halved (0.5 mM). Regarding lipophilicity (Table 1), a direct correlation between IC_50_ and CLog*P* values was not observed. It was observed, nonetheless, that the compounds with the lowest CLog*P* values were always amongst the least active (compounds **9**–**11**, with CLog*P* 0.975–1.421), and compound **24**, with a CLog*P* value (4.233) remarkably higher than the others, was also amongst the least active compounds. The CLog*P* of the most active compounds (**3**, **4**, **16**, **18**, and **23**) was in the range of 1.873–3.122. These results suggest that there is a certain requirement for lipophilicity to be in a certain range for bioactivity with a medium-high CLog*P* value needed (1.873–3.122), in agreement with Lipinskii’s rule of 5 [25]. In a previous work, a SAR study was carried out with hydrocinnamic acid (**2**) derivatives, where it was also found that CLog*P* values for active compounds against *C. campestris* growth were defined in this range [26]. Even so, adequate lipophilicity is not enough to induce inhibition, since other compounds fulfilling this requirement have shown moderate or poor activity, such as compound **1** or compounds **12**–**15**. Thus, specific structural features are required rather than only lipophilicity in the mechanism of the growth inhibition of *C. campestris*.

The SAR study revealed structural features with significance for the inhibitory activity of *C. campestris* growth. Firstly, regarding the degree of the unsaturation of the side chain, in comparison with *trans*-cinnamic acid (**1**), a decreased growth-inhibitory activity was observed in phenylpropiolic acid (**25**), with a triple bond, and a more pronounced, higher activity was observed in hydrocinnamic acid (**2**), characterized by a simple bond (Figure 3A–C). These results indicate that the nature of the bond between carbons 2 and 3 is a key factor influencing the growth-inhibitory activity. Although the electronic effect of the π electrons could be involved, Abe et al. [27] reported the importance of the geometry of the bond in these carbons for the phytotoxic activity against the growth of lettuce root, where the *cis* conformation was revealed to be key to the inhibitory activity. In our study, hydrocinnamic acid (**2**), with free rotation, could adopt a similar geometry, which would explain its higher activity in comparison with *trans*-cinnamic acid (**1**).

The inhibitory activity of derivative compounds with halogenated substituents on the aromatic ring (namely, compounds **18**–**21**) was increased when compared with *trans*-cinnamic acid (**1**). This finding agrees with a previous study where halogenated substituents increased the activity of the parent compound hydrocinnamic acid (**2**) against *C. campestris* growth [26] but disagrees with Nishikawa et al. [28], who report similar or slightly less activity for halogenated analogs when compared with the *cis*-isomer of cinnamic acid. In our work, the growth inhibitory effect of halogenated derivatives was dependent on the type of halogenated substituent and its position. On one side, when considering the substitution in the *para* position, the most phytotoxic compound was that containing the largest and more electronegative halogen Br (**21**, Figure 3D) > Cl (**20**) > F (**19**), confirming the activity of equivalent halogenated substituents in hydrocinnamic acid reported by Moreno-Robles et al. [26] and *cis*-cinnamic acid reported by Nishikawa et al. [28]. In addition, when comparing the results of compounds containing the same halogen in different positions, the one with a Cl atom in the *meta* position (compound **18**, IC_50_ = 461 µM) presented enhanced phytotoxicity when compared to the *para*-substituted isomer (compound **20**, IC_50_ = 955 µM). The effect on the activity levels was more pronounced regarding the position of the halogen (**18** and **20**) when compared with those compounds containing a different halogen atom in the same position (**19**–**21**) (Figure 2).

Hydroxyl groups in the aromatic ring had a negative effect on bioactivity, as can be observed by a comparison of the activity profile of compound **1** with those of the hydroxylated derivatives **6**–**9** (Figure 2). These compounds have the lowest CLog*P* values (Table 1), so this activity decrease could be related to lower lipophilicity and transport through the cell membranes, to a certain extent. There were differences in activity depending on the position of the hydroxyl group, with improved activity being found for the ortho derivative (compound **6**) when compared to the meta (compound **7**) or para (compound **8**) derivatives. These results agree with a previous study that evaluated the effects of hydroxyl substituents in the hydrocinnamic acid structure against *C. campestris* growth [26]. The increased activity of the hydroxylated derivative at the ortho position (**6**) may be due to its ability to cyclize and form coumarins. Indeed, a previous study reported phytotoxic activity for the coumarins scopoletin and umbelliferone against *C. campestris* growth [29]. Cyclization into scopoletin and umbelliferone occurs from cinnamic acid but requires an ortho alcohol [30], which could be the explanation for the increased activity found for the ortho hydroxylated derivative.

The methylation of the hydroxyl in compound **8** to give compound **12** caused an increase in bioactivity and lipophilicity, demonstrating the previous observation that the bioactivity was hindered by the presence of a hydroxyl group in the para position. On the other hand, similar inhibitory activity was obtained for compound **12** and for the parent compound *trans*-cinnamic acid (**1**). This result indicates a non-significant influence of this methoxy group on the growth-inhibitory activity, which did not generate significant changes in the lipophilicity of the compounds, as similar CLog*P* values were estimated (2.239 vs. 2.158). Previously, it was found that para-methoxy substitution had a decreasing effect on the activity when considering hydrocinnamic acid [26] and *cis*-cinnamic acid [28] as parent compounds.

Additionally, Moreno-Robles et al. [26] and Nishikawa et al. [28] found that *meta*-methoxy substitutions were more active than their respective parent compounds hydrocinnamic acid and *cis*-cinnamic acid. The bioactivity of methoxy substitution at the meta position on *trans*-cinnamic acid was not studied in our work, but the bioactivity of ferulic acid (**10**) with di-substitution of the aromatic ring, including a *meta*-methoxy group and a hydroxyl group at the *para* position, was decreased in comparison with the parent compound (**1**). This finding could indicate that the beneficial effect of the methoxy group in the *meta* is hindered due to the presence of an extra hydroxyl group in the *para* position. Accordingly, *trans*-sinapic acid (**11**), with two methoxy substituents and a hydroxyl group in the *para* position, and *trans*-caffeic acid (**9**), with two hydroxyl groups in the *para* and *meta* positions, showed diminished activity in comparison with the *trans*-cinnamic acid (**1**).

The *ortho*-trifluoromethylated compound (**15**) reduced the activity in comparison with *trans*-cinnamic acid (**1**), which agrees with the SAR study of Nishikawa et al. [28] on *cis*-cinnamic acid. The *para*-trifluoromethylated compound (**16**) improved the activity of the parent compound (**1**), which agrees with the SAR study by Moreno-Robles et al. [26] on hydrocinnamic acid against *C. campestris* growth, whereas an opposite case was observed in the SAR study by Nishikawa et al. [28] on *cis*-cinnamic acid against lettuce. The decreased activity of compound **15** could be due to the possibility that the trifluoromethyl group in the *ortho* position could be blocking the channeling of substituted cinnamic acid into coumarins.

The *ortho*-methylated compound **13** showed a decreased inhibitory activity compared to **1**. In contrast, Nishikawa et al. [28] reported that a methyl group at the *ortho* position in an analog of *cis*-cinnamic acid increased the inhibitory activity of lettuce growth. The para-methylation did not have an effect on the activity, as the activity of compound **14** was not significantly different from *trans*-cinnamic acid (**1**). Previously, it was reported that analogs of *cis*-cinnamic acid with methyl groups at the *meta* or *para* positions were slightly less active than the parent compound *cis*-cinnamic acid [28]. By contrast, the *para*-methylated compound 3-(*p*-tolyl)propionic acid had more pronounced activity than hydrocinnamic acid on *C. campestris* growth [26].

To evaluate the direct influence of the carboxylic acid group, the two esters methyl cinnamate (**23**) and benzyl cinnamate (**24**) were evaluated. The growth-inhibitory activity of methyl cinnamate (**23**) was significantly higher than that of the parent compound (**1**), while the inhibitory activity of benzyl cinnamate (**24**) was practically lost (Figure 2). In fact, compound **23** was the most active compound according to the IC_50_ values (Table 1), and its growth-inhibitory activity has been previously reported against lettuce, wheat, and annual ryegrass [31,32]. However, it was reported to have low growth inhibition activity against radish [33]. Moreover, structurally related methyl esters of aromatic compounds were active against the growth of different broomrape parasitic species [34].

The results obtained for compounds **1**, **23**, and **24** would allow some SAR conclusions regarding the influence of the carboxylic acid group on the reactivity to generate the inhibition of *C. campestris* growth. In the case of compound **1**, the acid group allowed moderate levels of inhibition. However, the improved activity shown by its methyl ester form (compound **23**), in comparison with compound **1**, would indicate that the carboxylic acid group causes the compound to have poorer activity due to probably higher acid properties. The difference in reactivity between compounds **1** and **23** is unlikely to be influenced by different lipophilicity given the similarity between the CLog*P* values calculated for compounds **1** and **23** (2.239 vs. 2.465). Compound **23** could have a better reactivity with the targets by a hydrolysis reaction, leading to a more phytotoxic byproduct such as methanol. In the case of compound **24**, with an additional aromatic ring next to the ester function, its leaving group has a pKa similar to compound **23**, so, chemically, they are expected to react in a similar way. However, its much higher lipophilicity (CLog*P* = 4.233, value out of range of Tice’s rule for post-emergence herbicides [35]) or its possible steric effects would make the molecule inactive.

The growth-inhibitory activity was also studied in cinnamaldehyde (**4**) and cinnamoyl alcohol (**5**), in which the carboxylic group was, respectively, reduced to aldehyde and alcohol. In both cases, the activity increased when compared to cinnamic acid (**1**, IC_50_ > 1000 µM), to an especially greater extent in the aldehyde case (**4**, IC_50_ = 408 μM). The increased inhibitory activity of *C. campestris* growth induced by the presence of the aldehyde group was also confirmed by the more pronounced activity of compound 3-phenylpropionaldehyde (**3**, IC_50_ = 497 μM) in comparison with the activity of hydrocinnamic acid (**2**, IC_50_ = 777 μM). In general, hydrocinnamic acid and its derivatives have been previously reported to have higher activity in *C. campestris* growth inhibition [26] than the inhibition activity of cinnamic acid and its derivatives shown in the present work. However, the increase in the activity of compound **4** in comparison with its parent compound **1** is much higher than the increase in the activity of compound **3** in comparison with its parent compound **2**. The importance of the double bond between carbons 2 and 3 could be of greater significance in the case of the mechanism of action in these aldehydes being different from the mechanism of action in the parent molecule.

**Figure 4:**
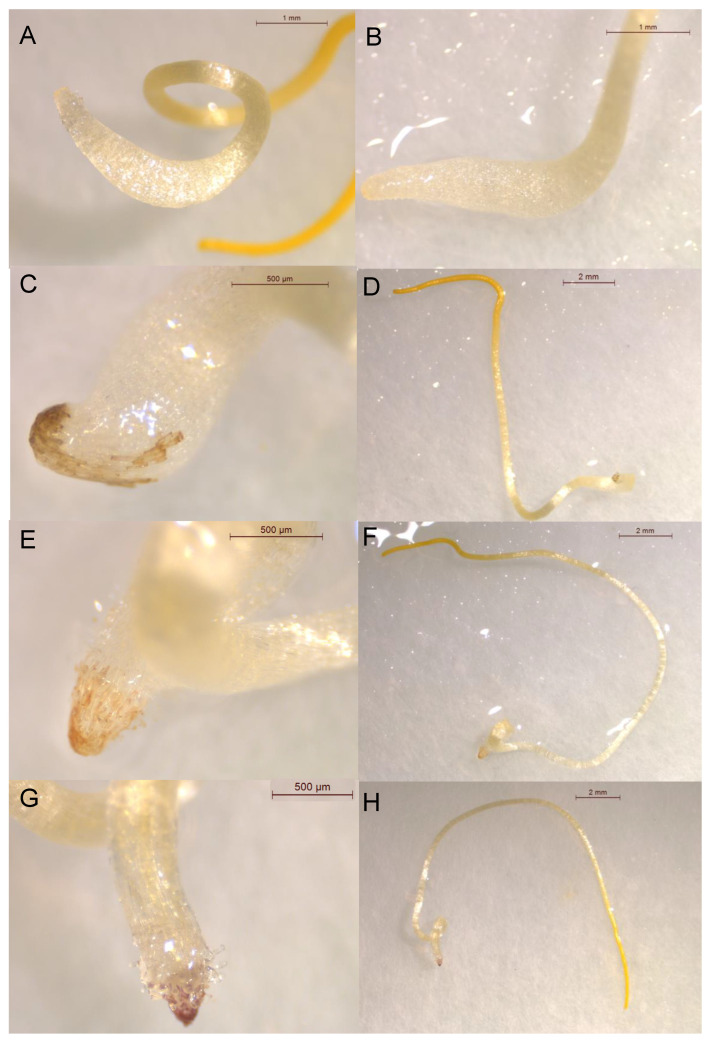
Photographs showing *C. campestris* root apices (**A**–**C**,**E**,**G**) and whole seedlings (**D**,**F**,**H**) treated with (**A**) a control or 1 mM treatments of (**B**) *trans*-cinnamic acid (**1**), (**C**,**D**) *trans*-caffeic acid (**9**), (**E**,**F**) *trans*-ferulic acid (**10**), and (**G**,**H**) *trans*-sinapic acid (**11**).

In addition to the growth-inhibitory activity, this study also identified compounds that induced the darkening of *C. campestris* root apices (Figure 4). The root apices of *C. campestris* seedlings treated with the cinnamic acid did not present any change in color in comparison with seedling controls (Figure 4A–B). Despite not having significant growth-reducing activity, intense darkening was observed in the root apices of seedlings treated with *trans*-caffeic acid (**9**) and *trans*-ferulic acid (**10**) (Figure 4C–F). The induction of dark coloration in *C. campestris* root apices by compounds **9** and **10** was previously reported by Moreno Robles et al. [29]. In addition to the confirmation of activity in compounds **9** and **10**, the present work identified the darkening-inducing activity of *trans*-sinapic acid (**11**) (Figure 4G–H). The compounds **9**, **10**, and **11** have in common the presence of an alcohol group in the *para* position. However, the alcohol group alone seems not to be the responsible feature for inducing darkening, because of the observed lack of activity in *trans*-*o*-coumaric acid (**6**), *trans*-*m*-coumaric acid (**7**), and *trans*-*p*-coumaric acid (**8**), with a hydroxyl group in the ortho, meta, and para positions, respectively. For the darkening-inducing activity, a minimum of two oxygenated substituents seems to be needed on the ring, and one of them is required to be an alcohol group, deduced from the fact that *trans*-3,4(methylendioxy)cinnamic acid (**22**) does not produce the darkening of root apices. An additional study with more substituents is recommended to further explore this hypothesis. A second hydroxy group (compound **9**) produces a pronounced coloration of a brown-black color in almost 100% of the seedlings treated (Figure 4C), while a second methoxy group (compound **10**) reduced the darkening-inducing activity, both in terms of the percentage of affected seedlings (50%) and also in the intensity of the coloration, shown by a lighter brown coloration in each seedling affected (Figure 4E). On the contrary, the presence of two methoxy groups in meta (compound **11**) produces an intense darkening response in 100% of the treated seedlings, observed as an intense brown-reddish coloration (Figure 4G). The three darkening-inducing compounds **9**, **10**, and **11** also have in common the lowest CLog*P* value (0.975, 1.421, and 1.204, respectively) among the 25 studied molecules. As mentioned before, when compared with compounds **9** and **11,** compound **10** had the lowest darkening-inducing activity and also the highest CLog*P* value, which could indicate that low lipophilicity could play a role in the effect of inducing the darkening of *C. campestris* roots.

## 3. Materials and Methods

### 3.1. Plant Material and Chemicals

Seeds of *C. campestris* were collected in the summer of 2022 from mature plants parasitizing pea plants at the Institute for Sustainable Agriculture (IAS-CSIC), Alameda del Obispo Research Center (Córdoba, southern Spain, coordinates 37.856 N, 4.806 W, datum WGS84). Dry seeds were separated from capsules by sifting with a 0.6 mm mesh sieve followed by winnowing with a fan. Seeds were stored dry in the dark at room temperature until use for this work.

*trans*-Cinnamic acid and its analogs were purchased from Sigma-Aldrich (St. Louis, MO, USA): *trans*-cinnamic acid (**1**, cat. n. C80857), hydrocinnamic acid (**2**, cat. n. 135232), 3-phenylpropionaldehyde (**3**, cat. n. 8045420100), *trans*-cinnamaldehyde (**4**, cat. n. 8025050250), *trans*-cinnamyl alcohol (**5**, cat. n. 108197), *trans*-*o*-coumaric acid (**6**, cat. n. H22809), *trans*-*m*-coumaric acid (**7**, cat. n. H23007), *trans*-*p*-coumaric acid (**8**, cat. n. C9008), *trans*-caffeic acid (**9**, cat. n. C0625), *trans*-ferulic acid (**10**, cat. n. 128708), *trans*-sinapic acid (**11**, cat. n. D7927), *trans*-4-methoxycinnamic acid (**12**, cat. n. M13807), *trans*-2-methylcinnamic acid (**13**, cat. n. 433101), *trans*-4-methylcinnamic acid (**14**, cat. n. M35800), *trans*-2-(trifluoromethyl)cinnamic acid (**15**, cat. n. 233080), *trans*-4-(trifluoromethyl)cinnamic acid (**16**, cat. n. 233099), *trans*-3-fluorocinnamic acid (**17**, cat. n. 290483), *trans*-3-chlorocinnamic acid (**18**, cat. n. 8413240010), *trans*-4-fluorocinnamic acid (**19**, cat. n. 222720), *trans*-4-chlorocinnamic acid (**20**, cat. n. C31600), *trans*-4-bromocinnamic acid (**21**, cat. n. 260975), *trans*-3,4-(methylenedioxy)cinnamic acid (**22**, cat. n. 146242), methyl *trans*-cinnamate (**23**, cat. n. 173282), benzylcinnamate (**24**, cat. n. 234214), and phenylpropiolic acid (**25**, cat. n. P31205).

### 3.2. Inhibitory Activity In Vitro Test against Cuscuta campestris Growth

A screening of the 25 compounds (**1**–**25**) described in Figure 1 was performed to identify phytotoxic activity against the growth of *C. campestris* seedlings. The germination of *C. campestris* seeds is inhibited by a thick seed coat that preserves seedbank viability in agricultural fields over time [2]. To promote *C. campestris* germination, the hard seed coat was eliminated by scarification with sulfuric acid for 45 min [36], followed by thorough rinses with sterile distilled water. Then, twenty scarified *C. campestris* seeds were placed using tweezers onto 5 cm-diameter filter paper discs inside 5.5 cm-diameter Petri dishes. All compounds were dissolved in dimethyl sulfoxide and then diluted to 1, 0.5, and 0.25 mM in MES 0.3 mM (2-(*N*-Morpholino) ethanesulfonic acid) (Sigma M-8250). The final concentration of dimethyl sulfoxide in all treatments was 1%. This was conducted for all compounds except for compounds **3**, **4**, and **5**, which were purchased in liquid form and dissolved directly into MES but supplemented with 1% of dimethyl sulfoxide to allow comparisons. Triplicate aliquots of 1 mL of each treatment were applied to filter paper discs containing the scarified *C. campestris* seeds. Triplicate aliquots of treatment only containing 1% of dimethyl sulfoxide and MES were used as a control. Treated *C. campestris* seeds were incubated in the dark at 23 °C for 5 days. The seedling length was measured in each of the five randomly chosen *C. campestris* seedlings for each of the three replicate filter paper discs per treatment. Seedling growth for each treatment was calculated in relation to the seedling growth of the corresponding control. In addition, notes were taken for each *C. campestris* seedling regarding whether the root apex had developed dark coloration. The percentage of seedlings that developed the darkening of root apices was calculated in each triplicated petri dish for each treatment.

### 3.3. Calculations and Statistical Analysis

All bioassays were performed using a completely randomized design. Percentage data were approximated to a normal frequency distribution by means of angular transformation. Then, percentage data were subjected to analysis of variance (two-way ANOVA). The significance of mean differences among treatments was evaluated by a two-sided Dunnett test at *p* < 0.05. Statistical analysis was performed using SPSS software 27 (SPSS Inc., Chicago, IL, USA). Compounds that reached inhibitions of 50% and that were active at more than one concentration were analyzed to determine their IC_50_ using GraphPad Prism v.5.00 software package (GraphPad Software, Inc., San Diego, CA, USA). The bioactivity data were fitted to a sigmoidal dose–response model with variable slope. The calculation of CLog*P* was performed using ChemOffice v20.1 (PerkinElmer, Waltham, MA, USA) using the appropriate tool in ChemDraw Professional [37].

## 4. Conclusions

In this study, *trans*-cinnamic acid and twenty four structural analogs were tested in vitro for their inhibition of the growth of *C. campestris*. The results showed that the most active compound was the methyl ester derivative of *trans*-cinnamic acid (**23**), with an IC_50_ of 331 μM. Nonetheless, other compounds exhibited close levels of inhibition (**3**, **4**, **16**, and **18**). Thus, both the different substitutions of the side chain and of the aromatic ring are important features to impart the inhibitory activity of *C. campestris* growth. In particular, key factors were the nature of the carbonyl group of the side chain and the presence of halogenated substituents on the aromatic ring. Compounds **9**, **10**, and **11** showed high levels of activity, inducing the darkening of the root apices. This activity was found to be related to the presence of methoxy and hydroxyl groups in the aromatic ring. This study allowed us to determine the structural features required for the inhibition of the growth of seedlings of *C. campestris* and to propose the methyl ester derivative of *trans*-cinnamic acid as a promising compound to design herbicides with enhanced activity against parasitic weeds.

## Figures and Tables

**Table 1:** Calculated CLog*P* and IC_50_ (μM) values of compounds **1**–**25**: ~1000, inhibition was close to 50% at the highest tested concentration; >1000, the compound did not induce 50% inhibition at the highest concentration tested but activity was significant; <250, the IC_50_ was lower than the lowest tested concentration.

	CLog*P*	IC_50_ (μM)		CLog*P*	IC_50_ (μM)		CLog*P*	IC_50_ (μM)
**1**	2.239	>1000	**11**	1.204	-	**21**	3.102	876
**2**	1.903	777	**12**	2.158	>1000	**22**	2.204	>1000
**3**	1.873	497	**13**	2.738	-	**23**	2.465	331
**4**	2.049	408	**14**	2.738	>1000	**24**	4.233	-
**5**	1.608	>1000	**15**	3.122	-	**25**	1.785	-
**6**	1.572	>1000	**16**	3.122	399			
**7**	1.572	-	**17**	2.382	~1000			
**8**	1.572	-	**18**	2.952	461			
**9**	0.975	-	**19**	2.382	~1000			
**10**	1.421	-	**20**	2.952	955			

## Data Availability

Not applicable.
